# Tradeoffs of microbial life history strategies drive the turnover of microbial-derived organic carbon in coastal saline soils

**DOI:** 10.3389/fmicb.2023.1141436

**Published:** 2023-03-23

**Authors:** Qi Ning, Lin Chen, Fang Li, Guixiang Zhou, Congzhi Zhang, Donghao Ma, Jiabao Zhang

**Affiliations:** ^1^Fengqiu Experimental Station of National Ecosystem Research Network of China, State Key Laboratory of Soil and Sustainable Agriculture, Institute of Soil Science, Chinese Academy of Sciences, Nanjing, China; ^2^College of Resources and Environment, Henan Agricultural University, Zhengzhou, China; ^3^University of Chinese Academy of Sciences, Beijing, China

**Keywords:** life history strategy, metagenome, metabolic traits, coastal saline soil, soil organic carbon, microbial carbon use efficiency

## Abstract

Stable soil organic carbon (SOC) formation in coastal saline soils is important to improve arable land quality and mitigate greenhouse gas emissions. However, how microbial life-history strategies and metabolic traits regulate SOC turnover in coastal saline soils remains unknown. Here, we investigated the effects of microbial life history strategy tradeoffs on microbial carbon use efficiency (CUE) and microbial-derived SOC formation using metagenomic sequencing technology in different salinity soils. The results showed that high-salinity is detrimental to microbial CUE and microbial-derived SOC formation. Moreover, the regulation of nutrients stoichiometry could not mitigate adverse effects of salt stress on microbial CUE, which indicated that microbial-derived SOC formation is independent of stoichiometry in high-salinity soil. Low-salinity soil is dominated by a high growth yield (Y) strategy, such as higher microbial biomass carbon and metabolic traits which are related to amino acid metabolism, carbohydrate metabolism, and cell processes. However, high-salinity soil is dominated by stress tolerance (S) (e.g., higher metabolic functions of homologous recombination, base excision repair, biofilm formation, extracellular polysaccharide biosynthesis, and osmolytes production) and resource acquisition (A) strategies (e.g., higher alkaline phosphatase activity, transporters, and flagellar assembly). These trade-offs of strategies implied that resource reallocation took place. The high-salinity soil microbes diverted investments away from growth yield to microbial survival and resource capture, thereby decreasing biomass turnover efficiency and impeding microbial-derived SOC formation. Moreover, altering the stoichiometry in low-salinity soil caused more investment in the A-strategy, such as the production of more *β*-glucosidase and *β*-*N*-acetyl-glucosaminidase, and increasing bacterial chemotaxis, which thereby reduced microbial-derived SOC formation. Our research reveals that shift the microbial community from S- and A- strategies to the Y-strategy is important to increase the microbial CUE, and thus enhance SOC turnover in coastal saline soils.

## 1. Introduction

Soil salinization seriously restricts the development of agricultural production. Of concern is that 25% of the total land is affected by salinity globally, and the saline-alkali soil area is growing at a rate of more than 1 million ha per year (Xia et al., 2019). The area of salinization soil reached 9.21 × 10^6^ ha, which accounted for 6.62% of the arable soil in China (Yang, 2008). Coastal saline soil is one of the primary types of saline-alkali soils, which is primarily distributed in coastal areas. The Yellow River Delta is a typical coastal saline soil area in China, and soil salinization has expanded rapidly from the coast to inland in this region during the past 20 years (Zhao et al., 2020), which has caused high soil salinity, poor soil structure, and low soil fertility (Xia et al., 2019). Saline soil is an important terrestrial carbon (C) sink with a high capacity for absorbing CO_2_ (Xie et al., 2009), which indicates an enormous potential for carbon sequestration. Therefore, enhancing the carbon sequestration capacity of coastal saline soils is highly important for mitigating greenhouse gas emissions and improving soil fertility.

The enhancement of soil organic carbon (SOC) is an effective measure to reduce salinity, improve fertility, and maintain sustainability in saline soils (Dong et al., 2022). Recently, numerous studies have revealed that microbial growth and its turnover products are major components of mineral-associated SOC (MASOC), which is a key fraction of stable SOC pools (Kallenbach et al., 2016; Liang et al., 2017; Sokol and Bradford, 2019; Georgiou et al., 2022). Living microbial biomass comprises only a small fraction of the total SOC, the persistent SOC accumulation primarily occurs through the rapid turnover of microbes to produce microbial-derived materials (Liang et al., 2017). The contribution of microbial-derived carbon to the SOC was reported to comprise more than 50% of the stable SOC and even up to 80% in some soils (Simpson et al., 2007; Sokol and Bradford, 2019). The process of mediation of the transformation of SOC by microorganisms is seriously affected by soil salinity. Typically, the toxic ions and high osmotic pressure in saline soils inhibit microbial growth and activity, which subsequently reduces the microbial biomass and metabolites, and is ultimately detrimental to SOC accumulation (Rath and Rousk, 2015). In addition, salinity affects the metabolic function and genes of microorganisms that are related to carbohydrate metabolism by altering the microbial community diversity and composition (Yang et al., 2021), which consequently exacerbates the loss of SOC. Microorganisms drive the generation and accumulation of SOC by catabolism and anabolism (Liang et al., 2017), and microbial carbon use efficiency (CUE) can be used to predict the balance between catabolic and anabolic metabolism (Rath and Rousk, 2015; Mo et al., 2021).

Recent literature proposed that the distinct prevailing microbial life history strategies (Y-A-S) could affect the microbial CUE and SOC accumulation under stress conditions (Malik et al., 2020). Specifically, high growth yield (Y) strategy microorganisms facilitate SOC accumulation and stabilization by increasing the turnover of microbial biomass. Resource acquisition (A) strategy microorganisms invest more energy in producing extracellular enzymes to acquire nutrient substrates, which contribute to the decomposition and losses of SOC. Stress tolerance (S) strategists divert resources away from growth in response to stress and therefore, reduce the microbial CUE and SOC formation. Metagenomic sequencing showed advantages in analyzing the microbial taxonomic community, as well as its functions and genes (Yang et al., 2021), and can provide a deeper understanding of the mechanisms of microbial-mediated SOC formation under stress habitats. Previous researchers have revealed the metabolic efficiency, microbial traits, and their underlying consequences on soil C dynamics under abiotic stress, such as cold (Feng et al., 2021) and drought stress (Li C. N. et al., 2022; Malik and Bouskill, 2022). However, the effect of salt stress on how microorganisms regulate their CUE and further affect SOC formation through the alternation of microbial life history strategies is rarely studied.

In addition, microbial CUE is also affected by homeostatic stoichiometry (Dong et al., 2022). Microorganisms require not only C sources for energy when converting substrate into microbial biomass but also an input of balanced nutrients, such as nitrogen (N) and phosphorus (P), to maintain a homeostasis of elemental stoichiometry (Mo et al., 2021). It is estimated that the microbial biomass C:N:P molar ratio of 60:7:1 is optimal for microbial growth (Cleveland and Liptzin, 2007). To reveal the microbial-mediated mechanisms of SOC formation under salt stress and imbalanced stoichiometry from the perspective of microbial life history strategy, we hypothesized that the microorganisms in highly saline soil are dominated by S-strategists, and microorganisms in low-salinity soil are dominated by Y-strategists; while the adjustment of stoichiometric relationships would mitigate the effect of salt stress (high- or low-salinity) on microbial CUE, and thereby alleviate the negative effect on soil SOC formation. In this study, coastal saline soils with different levels of salinity in the Yellow River Delta region were selected to analyze the differences in microbial CUE and microbial-derived SOC formation, and metagenomic sequencing technology was used to reveal the microbial trait-based strategies that drive SOC formation, aimed to provide new insights for carbon sequestration in coastal saline soils.

## 2. Materials and methods

### 2.1. Soil collection

Coast saline soils for this study were collected from the Yellow River Delta, which is located in the Kenli District, Dongying, Shandong Province, China (118°54′31″E, 37°39′58″N). This area has a continental monsoon climate, with an average annual temperature, precipitation, and evaporation of 12.3°C, 542 mm, and 1926 mm, respectively (Liu et al., 2020). The natural vegetation types in this area are halophytic plants, such as *Suaeda salsa* and *Phragmites australis*, and the farm field was planted with wheat (*Triticum aestivum*) for years. The soils are loamy clay that developed from the alluvial sediments and are classified as a Salic Fluvisol (Xia et al., 2019). We selected the typical *Suaeda salsa* community habitats and nearby farm fields as high-salinity (HS, soil salinity >8 g kg^−1^) and low-salinity (LS, soil salinity <2 g kg^−1^) soils, respectively. The two saline soils have four independent replicates. In specific, four plots of 30 m × 30 m were set in both *Suaeda salsa* community habitats and farm fields. Nine cores of bulk soils in each plot were sampled from the surface soil (0–15 cm) and mixed evenly into one soil sample. All soil samples without plant residues were air-dried and passed through a 2-mm mesh, and were used to determine background soil physicochemical properties. HS and LS soils are with 2,274 and 436 μS cm^−1^ of electrical conductivity (EC), and with 8.66 and 1.50 g kg^−1^ of total salt content, respectively. Other soil properties, such as the pH, C, N, P contents, C:N:P ratio, and particle composition, are shown in Table 1.

**Table 1:** Basic soil properties in high-salinity (HS) and low-salinity (LS) soil.

Soil property	Unit	HS soil	LS soil
pH_1:5_		7.67 ± 0.05	7.98 ± 0.05
EC_1:5_	μS cm^−1^	2,274 ± 18	436 ± 8
Total salinity	g kg^−1^	8.66 ± 0.10	1.50 ± 0.02
SOC	g kg^−1^	9.50 ± 0.17	11.28 ± 0.46
Total N	g kg^−1^	0.80 ± 0.04	1.06 ± 0.01
Total P	g kg^−1^	0.95 ± 0.03	1.05 ± 0.01
Available N	mg kg^−1^	52.71 ± 1.78	58.75 ± 4.73
Olsen-P	mg kg^−1^	28.70 ± 0.53	21.17 ± 0.77
C:N ratio		11.95 ± 0.57	10.62 ± 0.30
C:P ratio		10.07 ± 0.45	10.76 ± 0.50
N:P ratio		0.84 ± 0.07	1.01 ± 0.02
Clay	%	25.43 ± 0.33	32.95 ± 0.61
Silt	%	31.18 ± 0.46	35.50 ± 0.37
Sand	%	43.40 ± 0.14	31.55 ± 0.98

SOC, soil organic carbon; N, nitrogen; P, phosphorus.

### 2.2. Experimental design

The experiment included four treatments: addition with ^13^C-glucose, addition with ^13^C-glucose and NP nutrients, addition with NP nutrients, and non-addition (without ^13^C-glucose or NP nutrients). Thus, there are 32 microcosms in total (2 saline soils × 4 treatments × 4 replicates), and half of microcosms without ^13^C-glucose added were used to quantify ^13^C fractions derived from glucose but microbial metagenomic sequencing analysis was not performed. To eliminate the sampling and sieving interferences and activate the soil microbes, the air-dried soils were pre-incubated for a week at 25°C at 30% of water-holding capacity (WHC) before the experiments were conducted. Each microcosm was established by placing 200 g (dry weight) of soil into a 1 L glass jar, with the addition of 0.5 mg C per g dry soil per week using ^13^C-glucose (10 atom% labelled) to simulate root exudations (Sokol and Bradford, 2019). N (0.068 mg g^−1^ soil) and P nutrients (0.022 mg g^−1^ soil) were inputted to regulate the intrinsic stoichiometry of the soil. ^13^C-glucose and inorganic nutrients were added as an aqueous solution. An equivalent amount of deionized water was added to the control soil (without glucose or nutrients), and all the microcosms were maintained with a water content at 55% WHC.

Each glass jar contained two 50 ml vials, one with 20 ml of 1.0 M NaOH to absorb the CO_2_, and the other with 10 ml of deionized water to maintain a moist atmosphere (Auwal et al., 2021). An additional four blank jars that contained only water and NaOH were established to account for the headspace CO_2_. All the microcosms were sealed and incubated at 25°C in the dark. The NaOH vials were exchanged at 1, 3, 5, and 7 days to analyze the CO_2_ concentration and its isotopes and the microbial CUE in a week. Approximately 50 g of soil was collected after 1 week. One portion was stored at 4°C to analyze the dissolved organic C (DOC), microbial biomass C (MBC) and extracellular enzymes, while another portion was stored at −80°C for a metagenomics sequencing analysis. The remaining soil was incubated for 8 weeks and collected to analyze the MASOC, DOC, MBC, and extracellular enzymes.

### 2.3. Soil CO_2_-C, MBC, DOC, MASOC, and δ^13^C (‰)

The amount of CO_2_ was determined by titrating the NaOH solution with 1.0 M of standardized HCl. The stable ^13^C isotope ratios of CO_2_-C were determined as previously described (Auwal et al., 2021; Chen et al., 2022). Briefly, 10 ml of 1.0 M NaOH was added with 10 ml of 1.5 M BaCl_2_ to precipitate the carbonates. The BaCO_3_ precipitate was filtered, washed several times, and oven-dried at 60°C.

The soil MBC was measured using the chloroform fumigation-K_2_SO_4_ extraction method and calculated with a correction factor of 0.45 (Vance et al., 1987). The DOC was extracted from the non-fumigated soil sample. The contents of MBC and DOC were measured using a total organic carbon (TOC) analyzer (Analytik Jena AG, Jena, Germany), and the analysis of δ^13^C (‰) values of MBC and DOC used a persulfate digestion method according to Fang et al. (2020).

The soil samples were separated by a solution of sodium hexametaphosphate, and the dispersed soil was passed through a 53 μm sieve and collected as the MASOC fraction. The MASOC fraction was oven-dried at 60°C, and the inorganic C was removed with 6.0 M of HCl before the elemental and isotope analyses were conducted (Sokol and Bradford, 2019). The δ^13^C (‰) values of CO_2_-C, MBC, DOC, and MASOC were measured using an elemental analyzer-isotope ratio mass spectrometer (Thermo Fisher Scientific, Waltham, MA, United States).

### 2.4. Soil enzymes

The activities of *β*-glucosidase (GC), *N*-acetyl-*β*-glucosaminidase (NAG), and alkaline phosphatase (AKP) were quantified by measuring the rate at which the substrates were degraded using *p*-nitrophenyl-*β*-d-glucoside, *p*-nitrophenyl-*N*-acetyl-*β*-d-glucosaminide and *p*-nitrophenyl phosphate, respectively (Geisseler and Horwath, 2009). The specific enzyme activities were calculated as enzyme activities divided by the amount of MBC.

### 2.5. Metagenomic sequencing and bioinformatics

The soil microbial DNA was extracted using a FastDNA Spin Kit (MP Biomedicals, Santa Ana, CA, United States). The DNA concentrations and quality were measured using a NanoDrop spectrophotometer and 1% agarose gel electrophoresis, respectively. Qualified DNA was randomly fragmented to <500 bp by sonication with an S220 Focused-ultrasonicator (Covaris LLC, Woburn, MA, United States) for library construction. Sequencing was performed on an Illumina HiSeq 2,500 platform (Illumina, Inc., San Diego, CA, United States) using a 2 × 150 paired-end (PE) configuration. Quality control was conducted in Trimmomatic to remove low quality reads (quality value <30 or the presence of undetermined bases) and short reads (length < 50 bp) (Bolger et al., 2014). Clean reads were then *de novo* assembled into contigs by MEGAHIT (K-mer parameters: k-min 35, k-max 95, and k-step 20) (Li et al., 2015). The contigs >500 bp in length were selected for subsequent analysis. The open reading frames (ORFs) for the assembled contigs were predicted using Prodigal (Hyatt et al., 2010). The CD-HIT version 4.6 was used to construct a non-redundant gene catalogue with 95% identity and 90% coverage (Fu et al., 2012), and the gene counts and abundances in each sample were evaluated using Bowtie2 (Langmead and Salzberg, 2012) and SAMtools (Li et al., 2009). The assembled unigenes were compared with the NR database of NCBI using DIAMOND (blastp, evalue ≤1e-5) (Buchfink et al., 2015), and the LCA algorithm was used for taxonomic annotation. The functions of predicted protein sequences were annotated against the KEGG database using GhostKOALA (Kanehisa et al., 2016).

The theoretical partitioning of microbial life history strategies was proposed by Malik et al. (2020), and they used microbial functions as traits and assigned Y-A-S strategies to dominant populations in the community (Malik and Bouskill, 2022). The Y-strategists were defined as microbes that maximize their growth yield by enhancing central metabolism (e.g., C, N, and P metabolism) and biosynthesis (Malik et al., 2020; Li C. N. et al., 2022). The A-strategists were defined as microbes that invested more in functional traits that are associated with the complex substrates degradation, extracellular enzymes production, cell motility, transporters and siderophores (Malik et al., 2020; Shao et al., 2021). The S-strategists were defined as microbes that invested more in microbial traits that related to biomolecular damage repair, maintenance of cellular integrity, and osmolyte production (Malik et al., 2020; Malik and Bouskill, 2022). Next, the top 20 most abundant taxa at genus level with significant differences (*p* < 0.05) using an independent *t-*test between high- and low-salinity soils, were classified based on the Y-A-S strategies framework. The top 30 abundant functional categories at KEGG level 3 that differed significantly (*p* < 0.05) between high- and low-salinity soils were classified as Y-A-S strategies. In addition, specific genes were allocated to the Y-A-S strategies as described above. The abundance of microbial genes assigned to the Y-A-S strategies was transformed by a *Z*-score and visualized in heatmaps by the “pheatmap” package in R (v.4.1.1).

### 2.6. Calculation and statistics

The amounts of ^13^C-C pools (i.e., ^13^C-CO_2_, ^13^C-DOC, and ^13^C-MASOC) derived from glucose were estimated using the following equations (Arcand et al., 2017):


(1) $$F=\frac{\delta^{13}C_{\mathrm{sample}}-\delta^{13}C_{\mathrm{control}}}{\delta^{13}C_{\mathrm{glucose}}-\delta^{13}C_{\mathrm{control}}}$$

(2) $${}^{13}C-C_{\mathrm{pools}}(\mu g\;g^{-1})=F\times C_{\mathrm{pools}}$$

where F is the fraction (CO_2_-C, DOC, or MASOC) derived from the glucose, and δ^13^C_sample_ and δ^13^C_control_ are the corresponding δ^13^C (‰) value in the sample and control soil (without glucose), respectively. δ^13^C_glucose_ is the δ^13^C (‰) value of the ^13^C-labeled glucose. C_pools_ (μg g^−1^) is the amount of CO_2_-C, DOC, and MASOC in the soil samples amended with glucose, and ^13^C-C_pools_ is the amount of ^13^C-CO_2_, ^13^C-DOC, and ^13^C-MASOC derived from glucose.

The glucose-derived MBC (^13^C-MBC) and CUE were calculated as follows (Kallenbach et al., 2016; Fang et al., 2020):


(3) $$\delta^{13}\mathrm{MBC}=\frac{\delta^{13}C_{\mathrm{Fum}}\times C_{\mathrm{Fum}}-\delta^{13}C_{\mathrm{NFum}}\times C_{\mathrm{NFum}}}{C_{\mathrm{Fum}}-C_{\mathrm{NFum}}}$$

(4) $$\begin{array}{l} F_{\mathrm{MBC}}=\frac{\delta^{13}{\mathrm{MBC}}_{\mathrm{sample}}-\delta^{13}{\mathrm{MBC}}_{\mathrm{control}}}{\delta^{13}C_{\mathrm{glucose}}-\delta^{13}{\mathrm{MBC}}_{\mathrm{control}}} \end{array}$$

(5) $${}^{13}C-\mathrm{MBC}\;(\mu g\;g^{-1})=F_{\mathrm{MBC}}\times \mathrm{MBC}$$

(6) $$\mathrm{CUE}\;=\frac{{}^{13}C-\mathrm{MBC}}{{}^{13}C-\mathrm{MBC}{+}^{13}C-{\mathrm{CO}}_{2}}\times 100$$

where δ^13^MBC is the δ^13^C (‰) value of the MBC; δ^13^C_Fum_ and δ^13^C_NFum_ are the δ^13^C (‰) values of fumigated and non-fumigated extracts, respectively, and C_Fum_ and C_NFum_ represent the DOC concentrations (μg g^−1^) of the fumigated and non-fumigated extracts, respectively. δ^13^MBC_sample_ and δ^13^MBC_control_ are the corresponding δ^13^C (‰) value in the sample and control soil (without glucose), respectively. ^13^C-MBC and ^13^C-CO_2_ are the MBC and CO_2_ derived from glucose, respectively.

A one-way analysis of variance (ANOVA) with the least significant difference (LSD) test was performed in SPSS 19.0 (IBM, Inc., Armonk, NY, United States). Bar plots and boxplots were drawn in Origin2017 (OriginLab, Northampton, MA, United States). The relationships of microbial CUE and Y-A-S strategies were outlined using Pearson’s correlation analysis in the “corrplot” package in R, and significant differences between CUE and Y-A-S strategies were indicated by *p* < 0.05. The differences in genes between treatments with or without nutrient additions in low-salinity soil were analyzed using STAMP 2.1.3 (Parks et al., 2014).

### 2.7. Data availability statement

The raw data of metagenomic sequencing were deposited in the NCBI SRA database under accession number PRJNA891869.

## 3. Results

### 3.1. Allocation of ^13^C-glucose and specific enzyme activity

**Figure 1:**
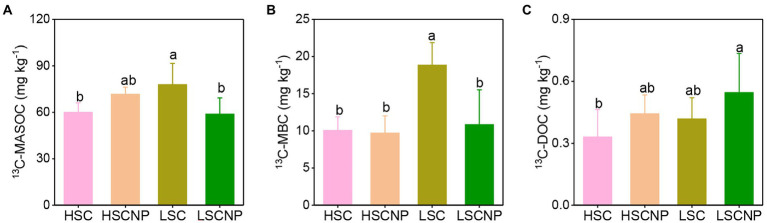
^13^C-Glucose-derived carbon in the pools of mineral-associated soil organic carbon (MASOC) **(A)**, microbial biomass carbon (MBC) **(B)**, and dissolved organic carbon (DOC) **(C)** after 56 days of incubation. Different letters indicate significant differences (*p* < 0.05). HSC, high-salinity soil added with ^13^C-glucose; HSCNP, high-salinity soil added with ^13^C-glucose and NP nutrients; LSC, low-salinity soil added with ^13^C-glucose; LSCNP, low-salinity soil added with ^13^C-glucose and NP nutrients.

The contents of ^13^C-MASOC and ^13^C-MBC were higher in low-salinity soil than in high-salinity soil. The addition or non-addition of nutrients showed no significant variation on the contents of ^13^C-MASOC, ^13^C-MBC, and ^13^C-DOC in high-salinity soil. Whereas, the addition of nutrients decreased ^13^C-MASOC and ^13^C-MBC by 24.5 and 42.5% in low-salinity soil, respectively (Figure 1).

The soil specific enzyme activity indicated the effectiveness of the substrate and the strategy of the microorganisms to acquire nutrients. In this study, the activity of AKP per unit of MBC was significantly higher in high-salinity than in low-salinity soil. The addition of nutrients resulted in a significant decrease in the specific activities of NAG and AKP in the early stage of incubation and a significant increase in the specific activities of GC and NAG during the later stage of incubation in low-salinity soil (Supplementary Figure S1).

### 3.2. Soil microbial CUE and the dominant genera involved in the Y-A-S strategies

**Figure 2:**
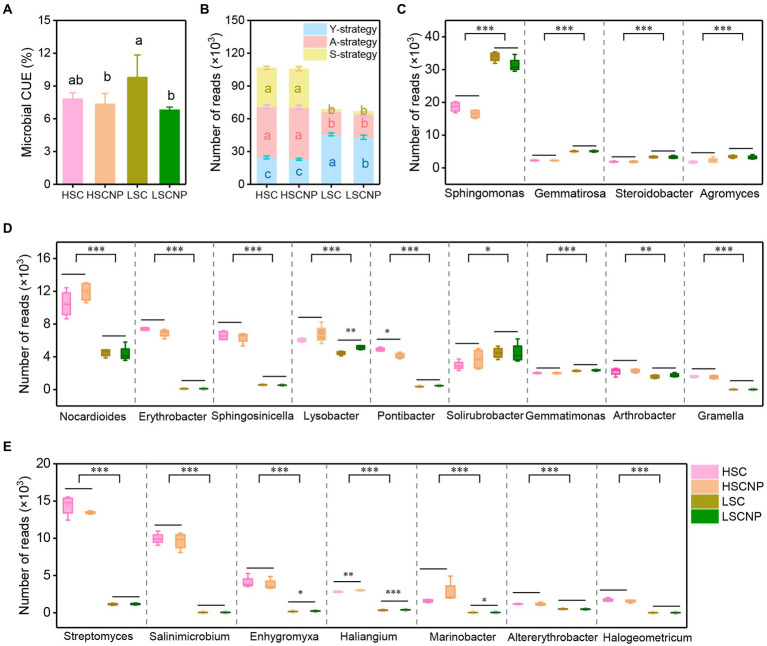
Soil microbial carbon use efficiency (CUE) after 7 days of incubation **(A)**. The sum of normalized read numbers of the top 20 abundant genera involved in the growth yield (Y-strategy), resource acquisition (A-strategy), and stress tolerance (S-strategy) **(B)**. The reads numbers of specific genes that were assigned to the Y-strategy **(C)**, A-strategy **(D)**, S-strategy **(E)**. Different letters indicate significant differences (*p* < 0.05), *p < 0.05, **p < 0.01. ***p < 0.001. HSC, high-salinity soil added with ^13^C-glucose; HSCNP, high-salinity soil added with ^13^C-glucose and NP nutrients; LSC, low-salinity soil added with ^13^C-glucose; LSCNP, low-salinity soil added with ^13^C-glucose and NP nutrients.

The microbial CUE was the highest (9.8%) in low-salinity soil without nutrients amended, which was significantly higher than that in high-salinity soil (7.8 and 7.3%). Moreover, the input of nutrients could not effectively increase the microbial CUE in high-salinity soil (Figure 2A).

The metagenome sequencing generated a total of 198.36 GB of raw data, and 189.89 GB of clean data were obtained after removing low-quality reads. 5,614,187 unique genes were identified, and 3,281,396 genes were phylogenetically classified into the bacteria (99.36%), micro-eukaryotes (0.04%), and archaea (0.60%). After excluding unassigned genes, 40.56% of genes presented a genus-level classification. The top 20 dominant genera (accounting for 27.23 ~ 45.55% of the total reads) were selected for in-depth analysis. We classified and summarized these top 20 genera according to the Y-A-S strategies (Supplementary Tables S1). The results showed that the A- and S-strategy microorganisms were dominant in high-salinity soil, while the abundance of Y-strategy microbes was the highest in low-salinity soil, followed by the A-strategy, with a small proportion of S-strategy microbes. The addition of nutrients resulted in a reduction in the abundance of Y-strategy microbes in low-salinity soil (*p* < 0.05) (Figure 2B). In particular, the genera *Sphingomonas, Gemmatirosa, Steroidobacter,* and *Agromyces*, which were involved in the Y-strategy, were prominently present in greater abundance in the low-salinity soil than in the high-salinity soil (*p* < 0.001) (Figure 2C). The abundance of seven genera, including *Nocardioides, Erythrobacter, Sphingosinicella, Lysobacter, Pontibacter, Arthrobacter,* and *Gramella*, that were associated with the A-strategy were found in remarkably higher levels in the high-salinity soil than in the low-salinity soil (*p* < 0.01) (Figure 2D). *Streptomyces, Salinimicrobium, Enhygromyxa, Haliangium, Marinobacter, Altererythrobacter,* and *Halogeometricum* assigned to the S-strategy were extremely significantly increased in the high-salinity soil (*p* < 0.001) (Figure 2E). Unexpectedly, the abundances of *Lysobacter, Enhygromyxa, Haliangium,* and *Marinobacter* that were involved in the A- and S-strategies increased with the input of nutrients to the low-salinity soil.

### 3.3. Microbial functional category involved in the Y-A-S strategies

**Figure 3:**
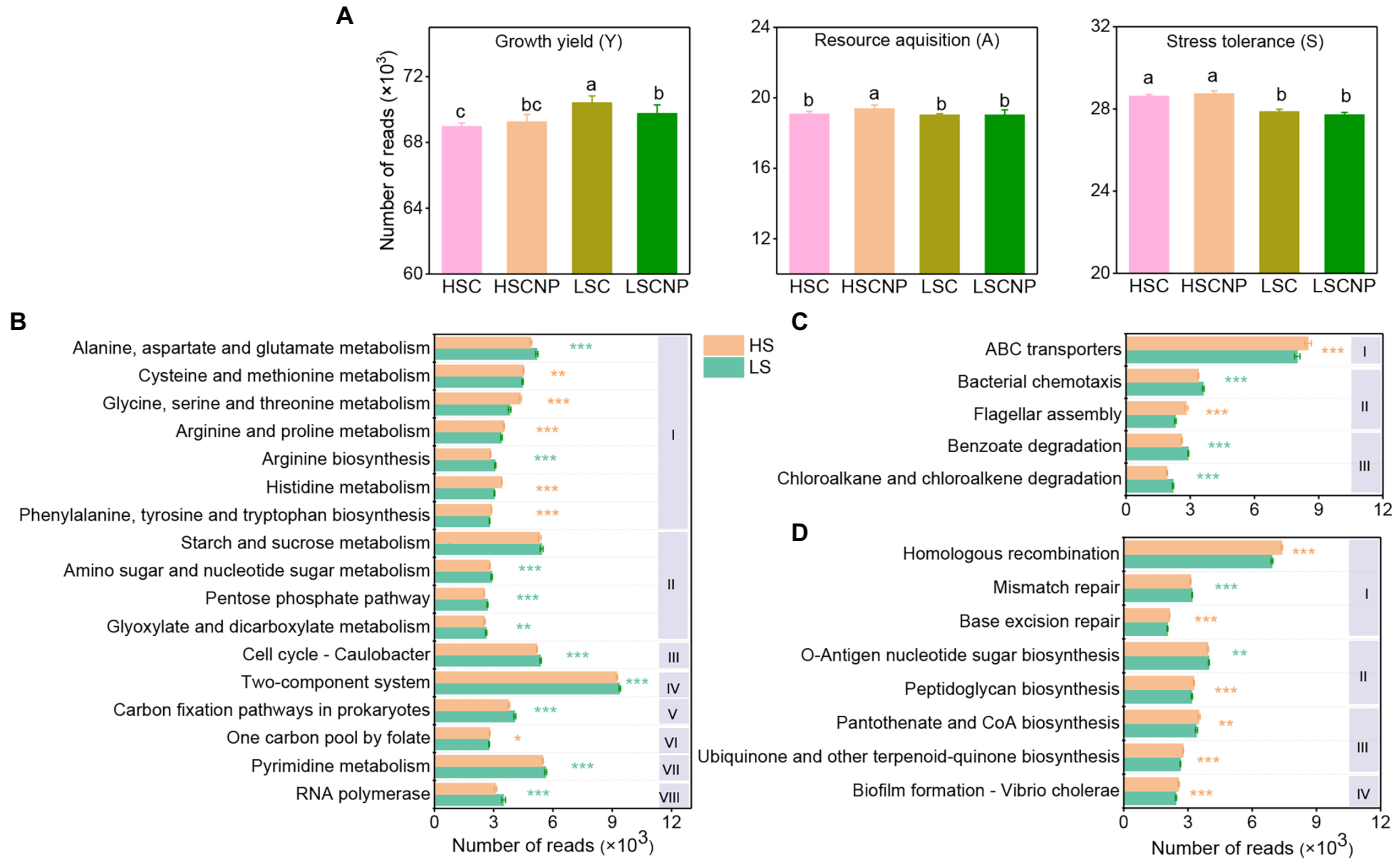
The reads numbers of functions related to the growth yield (Y-strategy), resource acquisition (A-strategy), and stress tolerance (S-strategy) according to KEGG annotations **(A)**. Specific KEGG functional categories assigned to the Y-strategy **(B)**, A-strategy **(C)**, and S-strategy **(D)**. The Roman numerals I, II, III, IV, V, VI, VII, and VII in subpanel **(B)** represent amino acid metabolism, carbohydrate metabolism, cell growth and death, signal transduction, energy metabolism, metabolism of cofactors and vitamins, nucleotide metabolism and transcription at the KEGG level 2, respectively. The Roman numerals I, II, and III in subpanel **(C)** represent membrane transport, cell motility and xenobiotics biodegradation and metabolism at the KEGG level 2, respectively. The Roman numerals I, II, III, and IV in subpanel **(D)** represent replication and repair, glycan biosynthesis and metabolism, metabolism of cofactors and vitamins and cellular community – prokaryotes, respectively. Orange asterisks indicate a higher abundance of functions in high-salinity soil, whereas green asterisks indicate a higher abundance of functions in low-salinity soil. Different letters indicate significant differences (*p* < 0.05). *p < 0.05, **p < 0.01, ***p < 0.001. HSC, high-salinity soil added with ^13^C-glucose; HSCNP, high-salinity soil added with ^13^C-glucose and NP nutrients; LSC, low-salinity soil added with ^13^C-glucose; LSCNP, low-salinity soil added with ^13^C-glucose and NP nutrients.

The top 30 abundant functional categories at KEGG level 3 with significant differences (*p* < 0.05) between high- and low-salinity soils were summarized in Supplementary Tables S2. Typically, the abundance of microbial functions involved in the growth yield was higher in low-salinity soil, but the addition of nutrients was detrimental to the increase of functions related to the Y-strategy in low-salinity soil, while the abundance of microbial functions involved in the A- and S-strategies were notably increased in high-salinity soils compared with low-salinity soils (*p* < 0.05) (Figure 3A). In particular, the functional categories related to amino acid metabolism (alanine, aspartate, and glutamate and arginine), carbohydrate metabolism (starch and sucrose metabolism, amino sugar and nucleotide sugar metabolism, pentose phosphate pathway, glyoxylate and dicarboxylate metabolism, carbon fixation pathways in prokaryotes) and cell processes (cell cycle-Caulobacter, pyrimidine metabolism and RNA polymerase) were especially reduced under high salt stress (Figure 3B). Instead, the microbial functions of ABC transporters and flagellar assembly were increased in high-salinity soil (Figure 3C). The microbial functions associated with S-strategy, such as homologous recombination, pantothenate and CoA biosynthesis, peptidoglycan biosynthesis, ubiquinone and other terpenoid-quinone biosynthesis, biofilm formation and base excision repair, were also significantly increased under high salt stress (Figure 3D). Furthermore, the input of nutrients to low-salinity soil remarkably increased the function of bacterial chemotaxis that were assigned to A-strategy, and decreased the functions of two-component system, pyrimidine metabolism, and arginine biosynthesis that were linked with the Y-strategy (Supplementary Figure S2).

### 3.4. Specific functional genes that were involved in Y-A-S strategies

**Figure 4:**
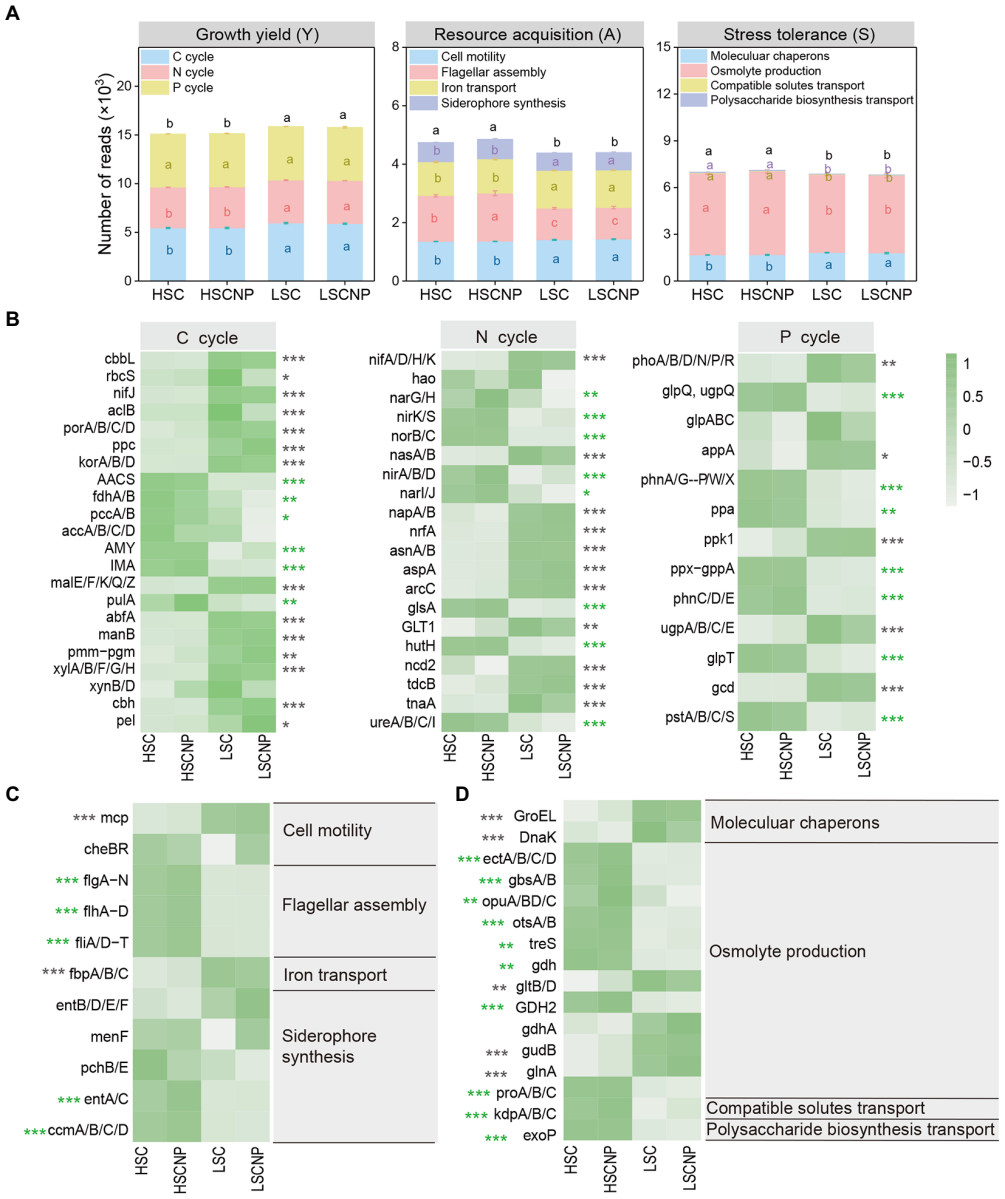
The normalized read numbers of genes assigned to growth yield (Y-strategy), resource acquisition (A-strategy), and stress tolerance (S-strategy) **(A)**. The heatmaps showed specific genes that corresponded to the Y-strategy **(B)**, A-strategy **(C)**, and S-strategy **(D)**, respectively. Green asterisks indicate a higher abundance of genes in high-salinity soil, whereas gray asterisks indicate a higher abundance of genes in low-salinity soil. To ensure that the heatmap was clear and concise, genes of the same gene cluster were combined and displayed together. More details are shown in Supplementary Data S1. Different letters indicate significant differences (*p* < 0.05). *p < 0.05, **p < 0.01, ***p < 0.001. HSC, high-salinity soil added with ^13^C-glucose; HSCNP, high-salinity soil added with ^13^C-glucose and NP nutrients; LSC, low-salinity soil added with ^13^C-glucose; LSCNP low-salinity soil added with ^13^C-glucose and NP nutrients.

In the present study, a total of 198 genes that encoded the functions involved in the Y-A-S strategies were detected. Most of them (116) were assigned to growth yield, and the abundance of these genes decreased with increasing salinity. A total of 54 and 28 genes that were assigned to resource acquisition and stress tolerance, respectively, were more abundant in high-salinity soil (Figure 4A and Supplementary Data S1). To ensure that the heatmap was clear and concise, genes of the same gene cluster were combined and displayed together. More details are shown in Supplementary Data S1. The genes that encoded functions in C (i.e., *cbbL, rbcS, nifJ, aclB, porA/B/C/D, ppc, korA/B/D, malE/F/K/Q/Z, abfA, manB, pmm-pgm, xylA/B/F/G/H, cbh,* and *pel*), N (i.e., *nifA/D/H/K, nasA/B, napA/B, nrfA, asnA/B, aspA, arcC, GLT1, ncd2, tdcB,* and *tnaA*), and P (i.e., *phoA/B/D/N/P/R, appA, ppk1, ugpA/B/C/E* and *gcd*) metabolism contributed to the increase in Y-strategy in low-salinity soil (Figure 4B). The genes that encoded flagellar assembly (*flg, flh,* and *fli* gene clusters) and siderophore synthesis (*entA/C* and *ccmA/B/C/D* genes) represented A-strategy under high salt stress (Figure 4C). The S-strategy related genes that encoded osmolyte production, such as *ectA/B/C/D, gbsA/B, opuA/B/C/D, otsA/B, treS, gdh, GDH2,* and *proA/B/C*, and genes that encoded compatible solute transport (*kdpA/B/C*) and polysaccharide biosynthesis transport (*exoP*) were strikingly higher (*p* < 0.001) in high-salinity soils than in low-salinity soils (Figure 4D). Moreover, the four genes that encoded C (*rbcS*), N (*nirK*), and P (*ugpE*) cycle and osmolyte production (*opuB/D*) decreased (*p* < 0.05) in the nutrient-added treatment of the low-salinity soil (Supplementary Figure S2). In addition, the abundance of genes that were assigned to Y-strategy, especially those involved in C and N cycle, were significantly (*p* < 0.05) positively correlated with microbial CUE. Whereas the genes encoding flagellar assembly and siderophore synthesis assigned to A-strategy were strongly negatively correlated with microbial CUE (Supplementary Figure S3).

## 4. Discussion

### 4.1. Microbial life history strategies mediated microbial CUE and microbial-derived SOC formation under high salt stress

The input of balanced nutrients did not exert a significant increase in MASOC and MBC in high-salinity soil, and the same trend was observed for microbial CUE (Figures 1, 2A), indicating that salt stress is detrimental to microbial growth and turnover, and the formation of microbial-derived SOC under high salt stress was independent from nutrient stoichiometry. A higher microbial CUE indicates that a greater proportion of substrate C was assimilated and converted toward microbial biomass synthesis, which potentially increased the generation and sequestration of stable SOC (Fang et al., 2020). In high-salinity soil, microorganisms sacrifice their growth to resist stress (Dong et al., 2022), which results in the decrease of microbial CUE and mineral-associated SOC content. Although previous results have shown that stoichiometry influenced the microbial CUE and SOC storage in soil that did not exhibit stress conditions (Mo et al., 2021), this study suggested that in saline soil, salt stress exerted stronger effects on CUE and MASOC, and the effects of stoichiometry were negligible, which is inconsistent with our hypothesis. These results were further confirmed by the trade-off of microbial life history strategies in high-salinity soil that involves stress tolerance combined with resource acquisition strategies.

Soil salinity altered the CUE due to its direct effect on microbial biomass, but it also indirectly affected the CUE by selecting different microbial life history strategies, which ultimately influenced the microbial-derived SOC formation. In high-salinity habitats, microbial taxa, functions, and genes that involved in S-strategy were prominently enriched. For example, several genera that increased (Figure 2), such as *Streptomyces, Salinimicrobium, Enhygromyxa, Haliangium, Marinobacter, Altererythrobacter,* and *Halogeometricum*, have been previously reported to be halotolerant microbes (Fudou et al., 2002; Cui et al., 2010; Gemperlein et al., 2018; Bachran et al., 2019; Etesami and Glick, 2020; Li et al., 2021). Under salt scenarios, these microbes have specific functional traits and genes to improve their resistance to salinity and could be divided into the following three categories (Figures 3, 4): (i) The microorganisms were capable of repairing biomolecular damage and maintaining cellular integrity, such as homologous recombination, base excision repair, and biofilm formation; (ii) the microorganisms induced the biosynthesis of extracellular polysaccharides, such as peptidoglycan biosynthesis and the *exoP* genes that are encoded; and iii) they produced osmolytes to maintain an osmotic balance by encoding the *ectA/B/C/D, gbsA/B, opuA/BD, otsA/B, treS, gdh, GDH2, proA/B/C,* and *kdpA/B/C* genes. Specifically, the ability to repair biomolecular damage was an essential metabolic trait under high-salinity stress, and similar results were found in other studies conducted in a saline environment (Wilson et al., 2004; Xu et al., 2022). The *exoP* gene is a polysaccharide biosynthetic transport protein that encodes biofilm formation. Biofilm formation and extracellular polysaccharide biosynthesis facilitate the concentration of resources to maintain microbial metabolic activity, which is consistent with previous studies on drought (Malik and Bouskill, 2022) and cold (Feng et al., 2021) stress. The production of osmolytes could maintain the cellular osmotic equilibrium and help to tolerate scarce resources, which is a very expensive energy consumption metabolic trait, and comes at the expense of reducing growth yield (Malik et al., 2020; Li C. N. et al., 2022). It takes a large amount of energy (30–110 ATP) to synthesize osmotic substances, which is much greater than those required to synthesize cell walls (30 ATP) (Chowdhury et al., 2011). This resulted in a significant metabolic burden on the microbes and reduces their energy for microbial growth. Some osmolytes are rich in C and N and can serve as energy sources after the stress has diminished (Malik and Bouskill, 2022). Ectoine, trehalose, glycine betaine, proline, and glutamate are the most common osmolytes that have been previously described (Malik et al., 2020; Malik and Bouskill, 2022). In addition, K^+^ uptake and transport and Na^+^/H^+^ antiporters are other metabolic methods to regulate osmotic pressure. Due to K^+^ is less toxic to the microbial community and metabolic function than Na^+^, microorganisms usually select the accumulation of K^+^ to reach an osmotic balance in a high-salinity environment (Rath and Rousk, 2015). All the microbial metabolic functions described that involved in the S-strategy are energy (C) expensive and result in a lower microbial CUE and microbial-derived SOC formation (Dong et al., 2022).

Strikingly, the A-strategy was also dominant in high-salinity soil, independent of nutrient input. The production of extracellular enzymes is a common way to increase resource capture. The specific enzyme activity per unit of MBC provides more sensitive indications for the microbial metabolic status and stability in extracellular enzymes activity, independently of changes in the MBC contents (Raiesi and Beheshti, 2014). Our research showed that salt stress results in a limitation of resources, and microorganisms produce more phosphatases and acquire P from soil organic matter to meet their growth under high salt stress (Supplementary Figure S1). Some specific genera, such as *Nocardioides, Erythrobacter, Sphingosinicella, Lysobacter, Pontibacter, Arthrobacter,* and *Gramella*, that involved in A-strategy were detected in high salt conditions (Figure 2). These taxa invest in the degradation of complex resources and secretion of hydrolytic enzymes to improve the availability of nutrients. For example, *Nocardioides* was reported to degrade xylan (Li et al., 2021). *Erythrobacter* is halophilic and can hydrolyze epoxides (Woo et al., 2007; Wang et al., 2021). *Sphingosinicella, Lysobacter,* and *Pontibacter* can produce hydrolytic enzymes, chitin-degrading enzymes, and glycoside hydrolase, respectively (Kato et al., 2009; Zhou et al., 2016; Fu et al., 2022). *Arthrobacter* and *Gramella* have ability to degrade recalcitrant polysaccharides, solubilize phosphate, and also obtain nutrients by cell motility (Bauer et al., 2006; Chen et al., 2006; Fan et al., 2014; Kabisch et al., 2014; Chen et al., 2019). Furthermore, resource acquisition markers, such as the microbial genes that encode ABC transporters (*ccmA/B/C/D*), flagellar assembly (*flg, flh,* and *fli* gene clusters), and siderophore biosynthesis (*entA/C*), were also widely found in high-salinity soils (Figure 4). The ABC transporters transport a variety of substrates using energy from ATP (Badri et al., 2009), and a greater investment in transporters contributed to the uptake of nutrient substrates by microorganisms under higher salt stress. Flagellar assembly was an indicator of the microbial capabilities of resource discovery (Malik et al., 2020). Previous studies confirmed that the *flg, flh,* and *fli* gene clusters that encode flagellar assembly are an expensive energy- consuming process and are intrinsically linked to biofilm formation (Wilkinson et al., 2011), which inevitably occurs at the expense of other physiological processes, such as growth yield. The genes *entA/C* that encode siderophore biosynthesis provide an increase in iron availability for microbial populations in highly saline soil. Iron plays an important role in biological systems under salt stress, such as those that catalyze enzymatic processes related to oxygen, hydrogen, and N, and are involved in DNA and RNA synthesis and repair processes (Ferreira et al., 2019).

Therefore, microbial physiology and the key pathways indicate that stress tolerance and resource acquisition are dominant life history strategies under high salt stress. Such strategies lead to resource reallocation as the microbes divert investments away from microbial growth yield and increase their investments in microbial survival and resource capture, thereby decreasing the efficiency of cellular growth and impacting the soil microbial-derived SOC formation.

### 4.2. Microbial life history strategies and stoichiometry affected the microbial-derived SOC formation in low-salinity soil

Compared with high-salinity soil, microbial CUE and MASOC were higher in low-salinity soil, demonstrating that soil microorganisms have a high growth yield strategy in the absence of salt stress, which is proved by the strongly positively correlations between microbial CUE and Y- strategy (Supplementary Figure S3). Specifically, the abundance of some microbial populations, such as *Sphingomonas, Gemmatirosa, Steroidobacter,* and *Agromyces*, increased when the salt stress was somewhat alleviated (Figure 2). These genera generally play a role in C and N metabolism, and thus, were assigned to the Y-strategy. *Sphingomona*s can metabolize various C sources, some species participate in N fixation and denitrification (Yang et al., 2016), and some strains produce salicylic acid to improve plant growth under salt stress (Etesami and Glick, 2020). Li W. J. et al. (2022) reported that species of *Gemmatirosa* were symbiotic flora and proliferated with the increase in the availability of water and organic matter. *Steroidobacter* possesses a strong ability to biodegrade sulfonamides, which are N-containing organic compounds (Zhang et al., 2021). As a member of the Actinobacteria, *Agromyces* was reported to be capable of N fixation (Marcos et al., 2019) and can decompose cellulolytic compounds by producing the *β*-glucosidase gene (Nevins et al., 2018). In addition, microbes can reduce the costs in stress tolerance and resource acquisition and increase their investment in central metabolism and biosynthesis, such as amino acid metabolism, carbohydrate metabolism, and cell processes, to maximize their growth yield in low-salinity habitats. For example, the increase in abundance of genes involved in the Calvin cycle, the reductive tricarboxylic acid cycle, starch catabolism, cellulose and hemicellulose catabolism, and pectin catabolism that related to Y-strategy are favored microbial C formation (Figure 4 and Supplementary Data S1). The Calvin cycle and reductive tricarboxylic acid cycle were the common C fixation pathways in farmland ecosystems (Huang et al., 2022). The higher abundance of the *cbbL* gene that is responsible for the Calvin cycle in low-salinity soil indicated a greater ability to fix CO_2_ than in high-salinity soil. Similar results in a previous study reported that salt stress inhibited the expression of activities of the Calvin cycle enzymes (Yu et al., 2011). Several genes, including *nifJ, aclB, porA/B/C/D, ppc,* and *korA/B/D*, that encode the reductive tricarboxylic acid cycle participate in the CO_2_ fixation metabolic pathway, which is favored to maximize the microbial growth yield owing to the low energy consumption of the reductive tricarboxylic acid cycle (Huang et al., 2022). In addition, starch, cellulose, hemicellulose, and pectin are easily taken up and metabolized by microorganisms (Chen et al., 2014), which thereby promotes their biomass growth.

However, the input of nutrients had negative effects on the microbial CUE and MASOC formation in low-salinity soil (Figure 1). The reduction in microbial CUE with the addition of nutrients suggested that a greater proportion of C was mineralized and released as the form of CO_2_ by microbes, while less C was converted to microbial biomass (Mo et al., 2021). The addition of N and P sources affected specific activities of GC, NAG, and AKP in low-salinity soil (Supplementary Figure S1). This probably occurred due to the N and P substrates that added were directly available for microbial growth, and there was no need to secrete additional extracellular enzymes during the early stage of incubation, which thereby decreased the activities of NAG and AKP. In contrast, during the later stage of incubation, the availability of substrates was reduced, and microorganisms needed to produce more GC and NAG to break down complex compounds into smaller substrates for uptake. Furthermore, the differences in life history traits also affected the microbial competition for C and nutrient flows (Mo et al., 2021). Our study showed that, compared with the lack of addition of nutrients, the metabolic traits and markers, such as *Lysobacter*, bacterial chemotaxis, and the *flgM* gene, that associated with the A-strategy were depleted in low-salinity soil that contained added nutrients. *Lysobacter* can produce a variety of lytic enzymes to degrade chitin, glucans, and proteins (Puopolo et al., 2018). Chemotaxis is a foraging strategy, which is primarily characterized by the use of flagella to swim and be motile, which enhances the bacterial uptake of nutrients and energy (Keegstra et al., 2022). The *flgM* gene, which encodes the flagellar assembly, has an important role in bacterial swimming and the formation of biofilms by regulating flagellar synthesis and flagellum numbers (Wilkinson et al., 2011). Taken together, the soil microbial community invested more C in the A-strategy and primarily through the secretion of extracellular enzymes and bacterial chemotaxis to enhance their resource capture ability, thereby influencing microbial-derived SOC formation.

## 5. Conclusion

Overall, the tradeoff of microbial life history strategies in the soils of different salinity affected the microbial CUE and microbial-derived SOC formation. Microbes invested more energy and resources in S- and A- strategies at the expense of growth yield in high-salinity habitats. Specifically, microorganisms increased their metabolic traits that are involved in biomolecular damage repair, extracellular polysaccharide biosynthesis, and osmolyte production and transport to improve their resistance to salinity. They also increased the synthesis of siderophores and flagellar assembly to acquire nutrient resources. In contrast, microbes primarily invested in a Y-strategy with a small proportion in A-strategy in low-salinity soil. The adjustment of stoichiometry had negligible effects on the promotion of microbial CUE and microbial-derived SOC formation in high-salinity soil. Moreover, the alteration of soil stoichiometry by nutrient addition increased the microbial metabolic traits in the shift to A-strategy in low-salinity soil, which was also detrimental to microbial-derived SOC formation. Therefore, for saline soil, the most important thing is to alleviate salt stress rather than regulate stoichiometry, so as to shift the microbial life strategy from S- and A- strategies to the Y-strategy, thereby increasing the microbial CUE and enhancing microbial-derived SOC formation.

## Data availability statement

The datasets presented in this study can be found in online repositories. The names of the repository/repositories and accession number(s) can be found in the article/Supplementary material.

## Author contributions

JZ, QN, and LC jointly developed the overall approach. QN and LC carried out the experimental work, did metagenomic sequencing data analysis, and manuscript preparation. FL, GZ, CZ, DM, and JZ revised the manuscript. All authors contributed critically to the drafts and gave final approval for publication.

## Funding

This work was jointly supported by the Strategic Priority Research Program of the Chinese Academy of Sciences (XDA24020104 and XDA28020203), the National Key Research and Development Program of China (2022YFD1500203 and 2022YFD1500401), the National Natural Science Foundation of China (42177332), the China Agriculture Research System (CARS-03-15 and CARS-52), and the Jiangsu Provincial Postdoctoral Science Foundation (2021Z241).

## Conflict of interest

The authors declare that the research was conducted in the absence of any commercial or financial relationships that could be construed as a potential conflict of interest.

## Publisher’s note

All claims expressed in this article are solely those of the authors and do not necessarily represent those of their affiliated organizations, or those of the publisher, the editors and the reviewers. Any product that may be evaluated in this article, or claim that may be made by its manufacturer, is not guaranteed or endorsed by the publisher.

## Supplementary material

SUPPLEMENTARY FIGURE S1 Soil specific enzyme activities per unit of microbial biomass (mg product g−1 MBC) in day 7 (**A-C**) and day 56 (**D-F**). Different letters indicate significant differences (*P* < 0.05). HSC, high-salinity soil added with 13C-glucose; HSCNP, high-salinity soil added with 13C-glucose and NP nutrients; LSC, low-salinity soil added with 13C-glucose; LSCNP low-salinity soil added with 13C-glucose and NP nutrients.

SUPPLEMENTARY FIGURE S2 Genes with significant differences between treatments with or without nutrient additions in low salinity soil. LSC, low-salinity soil added with 13C-glucose; LSCNP low-salinity soil added with 13C-glucose and NP nutrients.

SUPPLEMENTARY FIGURE S3 Pearson correlations of microbial CUE and Y-A-S strategies (**A**) and the relationships between Y-A-S strategies based on related genes classification and microbial CUE (**B**). * mean *P* < 0.05. Y-strategy, growth yield; A-strategy, resource acquisition; S-strategy, stress tolerance.
