# Supplementary figures and images for: Tradeoffs of microbial life history strategies drive the turnover of microbial-derived organic carbon in coastal saline soils
Source: Front Microbiol. 2023 Mar 23;14:1141436. doi: 10.3389/fmicb.2023.1141436 (PMC10076556; doi:10.3389/fmicb.2023.1141436)

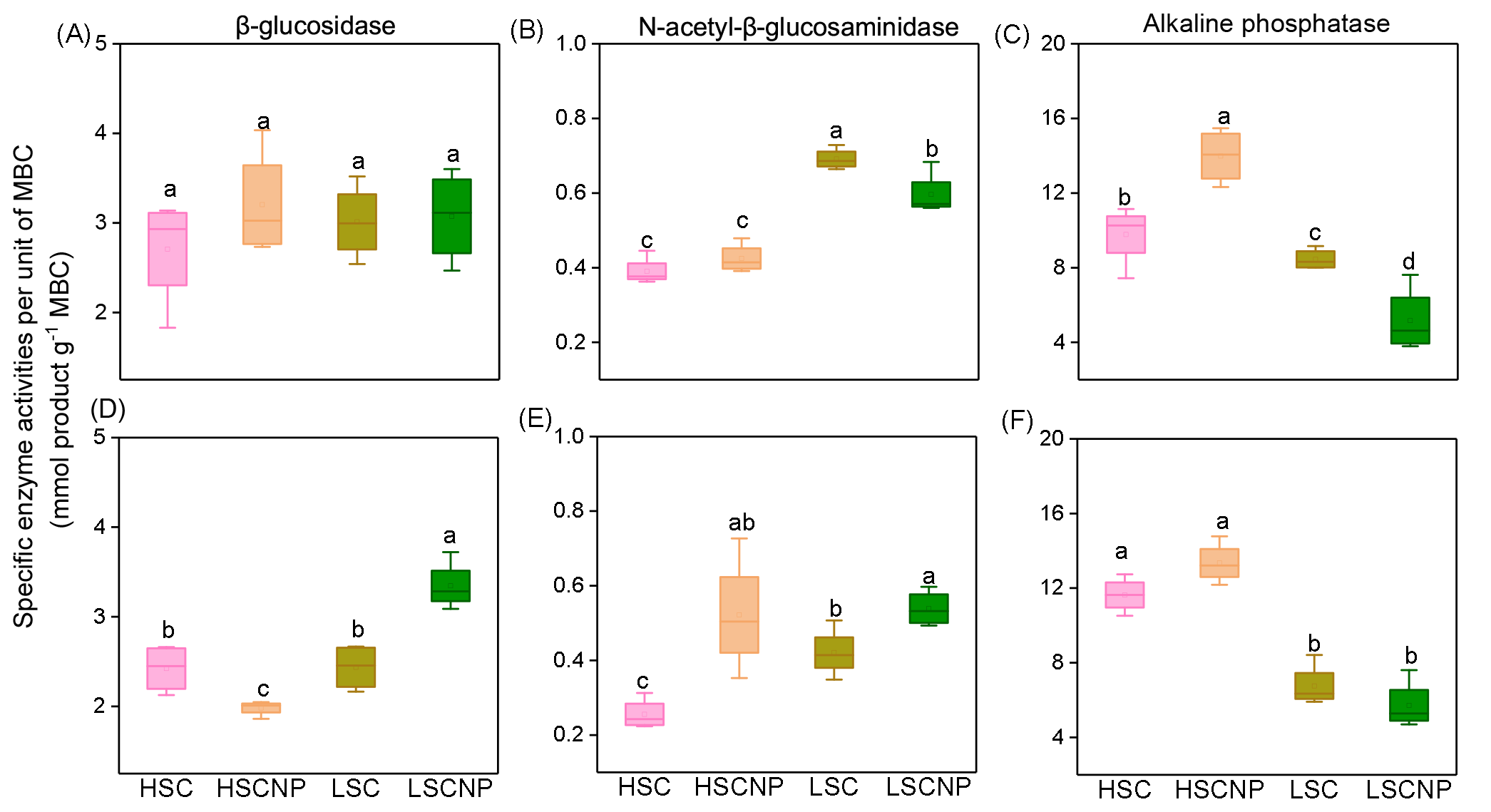

Supplement: SUPPLEMENTARY FIGURE S1 — Soil specific enzyme activities per unit of microbial biomass (mg product g−1 MBC) in day 7 (A-C) and day 56 (D-F). Different letters indicate significant differences (P < 0.05). HSC, high-salinity soil added with 13C-glucose; HSCNP, high-salinity soil added with 13C-glucose and NP nutrients; LSC, low-salinity soil added with 13C-glucose; LSCNP low-salinity soil added with 13C-glucose and NP nutrients. [file Image_1.tif]

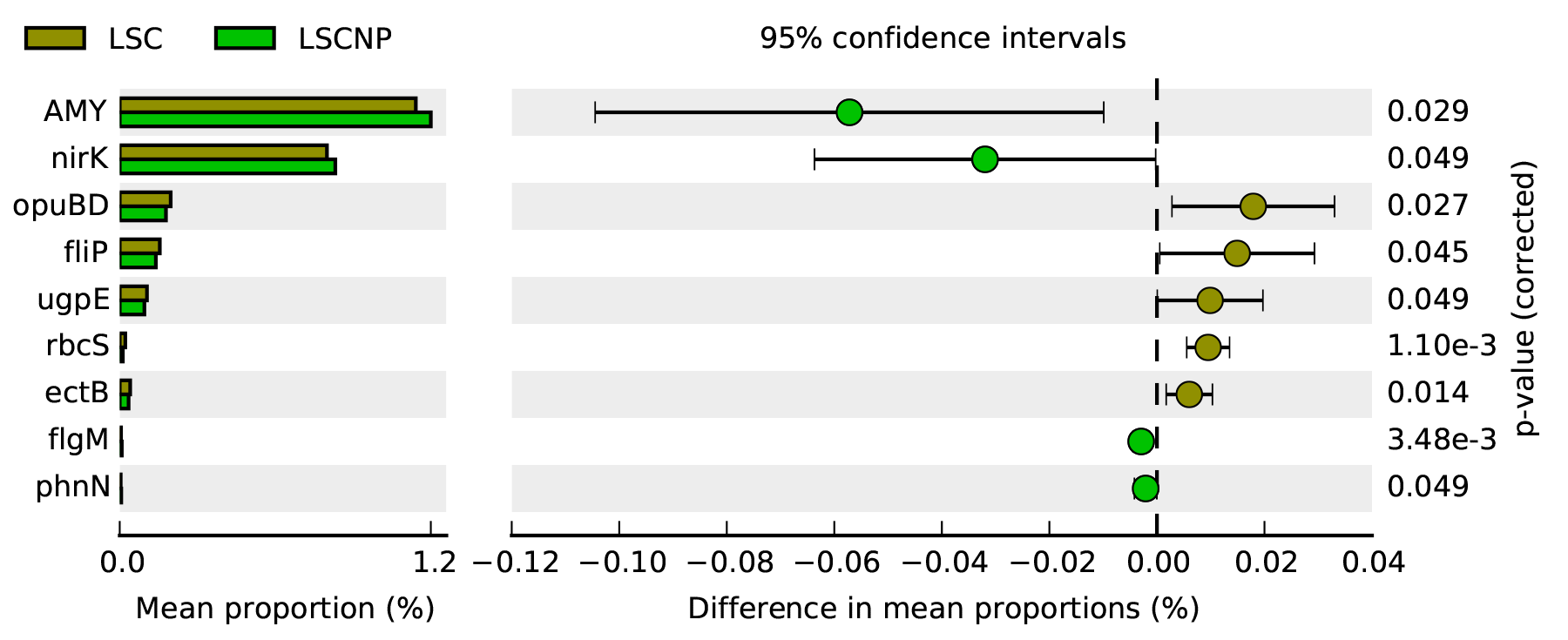

Supplement: SUPPLEMENTARY FIGURE S2 — Genes with significant differences between treatments with or without nutrient additions in low salinity soil. LSC, low-salinity soil added with 13C-glucose; LSCNP low-salinity soil added with 13C-glucose and NP nutrients. [file Image_2.tif]

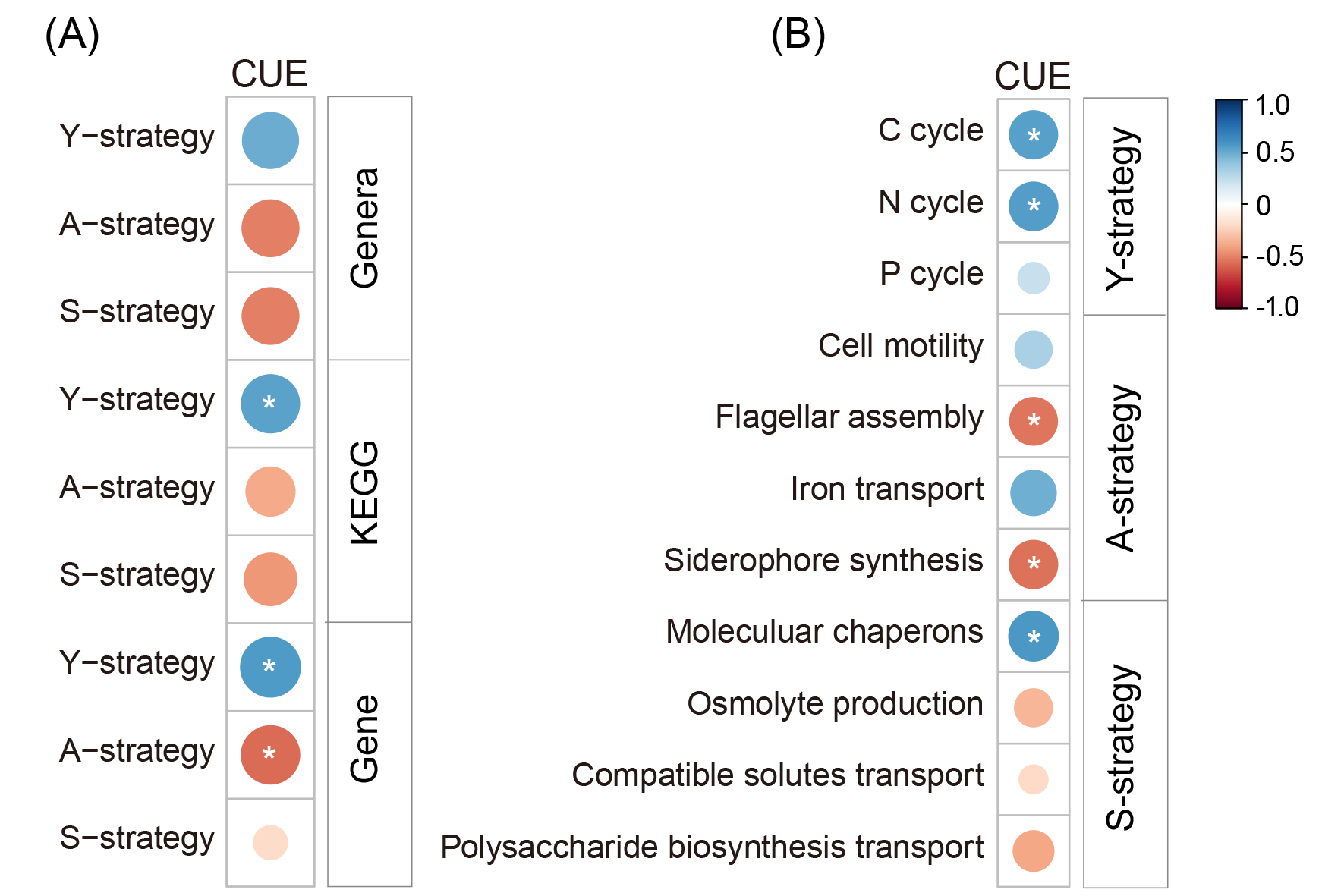

Supplement: SUPPLEMENTARY FIGURE S3 — Pearson correlations of microbial CUE and Y-A-S strategies (A) and the relationships between Y-A-S strategies based on related genes classification and microbial CUE (B). * mean P < 0.05. Y-strategy, growth yield; A-strategy, resource acquisition; S-strategy, stress tolerance. [file Image_3.tif]
