# Supplementary material for: Tradeoffs of microbial life history strategies drive the turnover of microbial-derived organic carbon in coastal saline soils
Source: Front Microbiol. 2023 Mar 23;14:1141436. doi: 10.3389/fmicb.2023.1141436 (PMC10076556; doi:10.3389/fmicb.2023.1141436)
Supplement: Supplementary file 4 [file Table_1.docx]

Table S1 Top 20 abundant genera and their functions and microbial life strategies

| Genus | Strategy | Function | Reference |
| --- | --- | --- | --- |
| Sphingomonas | Y | Nitrogen fixation and denitrification | (Gao et al., 2022a; Thiem et al., 2018) |
| Gemmatirosa | Y | Symbiotic flora | (Li et al., 2022b) |
| Steroidobacter | Y | Denitrification | (Xun et al., 2021; Yang et al., 2016) |
| Agromyces | Y | Nitrogen fixation | (Marcos et al., 2019) |
| Nocardioides | A | Degrade xylan | (Li et al., 2021) |
| Erythrobacter | A/S | Hydrolyze epoxides/ Halotolerant | (Wang et al., 2021; Woo et al., 2007) |
| Sphingosinicella | A | Possesses hydrolytic enzymes | (Kato et al., 2009) |
| Lysobacter | A | Produce chitin-degrading enzymes | (Fu et al., 2022) |
| Pontibacter | A | Glycoside Hydrolase | (Zhou et al., 2016) |
| Solirubrobacter | A | Utilize polysaccharides and biopolymers | (Jeewani et al., 2021) |
| Gemmatimonas | A | Decompose polyaromatic C | (Li et al., 2017) |
| Arthrobacter | A | Recalcitrant C decomposer, phosphate-solubilizing | (Chen et al., 2019; Chen et al., 2006; Fan et al., 2014) |
| Gramella | A | Polysaccharide utilization, motility | (Bauer et al., 2006; Kabisch et al., 2014) |
| Streptomyces | S | Saline-alkali tolerance | (Etesami and Glick, 2020; Gao et al., 2022b) |
| Salinimicrobium | S | Halotolerant microbes | (Bachran et al., 2019) |
| Enhygromyxa | S | Halotolerant | (Gemperlein et al., 2018) |
| Haliangium | S | Obligate halophile | (Fudou et al., 2002) |
| Marinobacter | S | Typical salt-tolerant bacteria, moderately halophilic | (Bachran et al., 2019; Li et al., 2021; Liu et al., 2019) |
| Altererythrobacter | S | Salt-tolerant bacterial strains | (Li et al., 2021) |
| Halogeometricum | S | Halophilic archaea | (Cui et al., 2010) |

Note: Y, growth yield strategy; A, resource acquisition strategy; S, stress tolerance strategy.
