# Supplementary material for: Tradeoffs of microbial life history strategies drive the turnover of microbial-derived organic carbon in coastal saline soils
Source: Front Microbiol. 2023 Mar 23;14:1141436. doi: 10.3389/fmicb.2023.1141436 (PMC10076556; doi:10.3389/fmicb.2023.1141436)
Supplement: Supplementary file 5 [file Table_2.docx]

Table S2 Significantly different KEGG functions and related strategies in high and low salinity soils.

| KEGG_Level 3 | Strategy | Average gene counts | | | | Reference |
| --- | --- | --- | --- | --- | --- | --- |
|  |  | HSC | HSCNP | LSC | LSCNP |  |
| RNA polymerase | Y | 3099 b | 3096 b | 3551 a | 3446 a | (Malik et al., 2020) |
| Pyrimidine metabolism | Y | 5471 c | 5497 c | 5669 a | 5589 b | (Li et al., 2022a) |
| One carbon pool by folate | Y | 2784 ab | 2799 a | 2777 ab | 2753 b | (Li et al., 2022a) |
| Carbon fixation pathways in prokaryotes | Y | 3757 b | 3774 b | 4097 a | 4034 a | (Li et al., 2022a) |
| Two-component system | Y | 9220 c | 9250 c | 9411 a | 9339 b | (Li et al., 2022a) |
| Cell cycle - Caulobacter | Y | 5192 b | 5168 b | 5387 a | 5358 a | (Li et al., 2022a) |
| Glyoxylate and dicarboxylate metabolism | Y | 2511 b | 2551 b | 2626 a | 2613 a | (Malik et al., 2020) |
| Pentose phosphate pathway | Y | 2523 b | 2520 b | 2695 a | 2670 a | (Malik et al., 2020) |
| Amino sugar and nucleotide sugar metabolism | Y | 2804 b | 2795 b | 2903 a | 2898 a | (Malik et al., 2020) |
| Starch and sucrose metabolism | Y | 5311 b | 5345 ab | 5442 a | 5408 ab | (Malik et al., 2020) |
| Phenylalanine, tyrosine and tryptophan biosynthesis | Y | 2876 a | 2890 a | 2790 b | 2789 b | (Li et al., 2022a) |
| Histidine metabolism | Y | 3403 a | 3416 a | 3046 b | 3033 b | (Li et al., 2022a) |
| Arginine biosynthesis | Y | 2814 c | 2842 c | 3103 a | 3051 b | (Li et al., 2022a) |
| Arginine and proline metabolism | Y | 3512 a | 3514 a | 3417 b | 3376 b | (Malik and Bouskill, 2022) |
| Glycine, serine and threonine metabolism | Y | 4343 a | 4365 a | 3826 b | 3790 b | (Malik and Bouskill, 2022) |
| Cysteine and methionine metabolism | Y | 4489 ab | 4527 a | 4459 b | 4461 b | (Malik et al., 2020) |
| Alanine, aspartate and glutamate metabolism | Y | 4851 b | 4902 b | 5213 a | 5158 a | (Malik et al., 2020) |
| Chloroalkane and chloroalkene degradation | A | 1901 b | 1922 b | 2204 a | 2194 a | (Malik et al., 2020) |
| Benzoate degradation | A | 2586 c | 2622 b | 2913 a | 2907 a | (Malik et al., 2020) |
| Flagellar assembly | A | 2766 b | 2870 a | 2298 c | 2313 c | (Malik et al., 2020) |
| Bacterial chemotaxis | A | 3377 c | 3386 c | 3584 b | 3638 a | (Li et al., 2022a) |
| ABC transporters | A | 8439 a | 8575 a | 8024 b | 7974 b | (Malik and Bouskill, 2022) |
| Biofilm formation - Vibrio cholerae | S | 2546 a | 2579 a | 2456 b | 2418 b | (Malik and Bouskill, 2022) |
| Ubiquinone and other terpenoid-quinone biosynthesis | S | 2767 a | 2770 a | 2641 b | 2642 b | (Zhao et al., 2022) |
| Pantothenate and CoA biosynthesis | S | 3493 ab | 3526 a | 3417 bc | 3375 c | (Wang et al., 2020) |
| Peptidoglycan biosynthesis | S | 3281 a | 3269 a | 3193 b | 3163 b | (Malik et al., 2020) |
| O-Antigen nucleotide sugar biosynthesis | S | 3944 ab | 3906 b | 3968 a | 3980a | (Malik et al., 2020) |
| Base excision repair | S | 2129 a | 2142 a | 2052 b | 2030 b | (Malik et al., 2020) |
| Mismatch repair | S | 3102 b | 3130 b | 3201 a | 3190 a | (Malik et al., 2020) |
| Homologous recombination | S | 7356 a | 7410 a | 6934 b | 6914 b | (Malik et al., 2020) |

Note: Y, growth yield strategy; A, resource acquisition strategy; S, stress tolerance strategy. Different letters in a same row indicate significant difference at 0.05 level. HSC, high-salinity soil added with ^13^C-glucose; HSCNP, high-salinity soil added with ^13^C-glucose and NP nutrients; LSC, low-salinity soil added with ^13^C-glucose; LSCNP low-salinity soil added with ^13^C-glucose and NP nutrients.
